# Socioeconomic deprivation does not affect survival outcomes in patients with mantle cell lymphoma: evidence from a German nationwide study

**DOI:** 10.1038/s41408-026-01658-w

**Published:** 2026-10-30

**Authors:** Susanne von Kroge, Sabrina Klotz, Hanna Walk, Carsten Bokemeyer, Fabian Tetzlaff, Henrik Kusche, the “LYSED” study group, Nadia Obi, Olaf von dem Knesebeck, Mareile Beernink, Christopher Gundler, Frank Ückert, Katharina Schulze, Jobst Augustin, Regina Semmelhaack, Anastasia Sassenberg-Gummert, Frederik Peters

**Affiliations:** 1 Department of Oncology, Hematology and Bone Marrow Transplantation with Section Pneumology, University Cancer Center Hamburg, University Medical Center Hamburg-Eppendorf, Hamburg, Germany; 2 Division of Social Determinants of Health, Department of Epidemiology and Health Monitoring, Robert Koch Institute, Berlin, Germany; 3 Hamburg Cancer Registry, Ministry of Science, Research and Equality, Free and Hanseatic City of Hamburg, Hamburg, Germany; 4 Institute for Occupational and Maritime Medicine, University Medical Center Hamburg-Eppendorf, Hamburg, Germany; 5 Department of Medical Sociology, University Medical Center Hamburg-Eppendorf, Hamburg, Germany; 6 Institute for Applied Medical Informatics, University Medical Center Hamburg-Eppendorf, Hamburg, Germany; 7 Institute for Health Services Research in Dermatology and Nursing, University Medical Center Hamburg-Eppendorf, Hamburg, Germany; 8 DAK Gesundheit, Hamburg, Germany

**Keywords:** B-cell lymphoma, Risk factors

## Abstract

Socioeconomic status (SES) is well recognized as an important determinant of cancer incidence and survival. However, its impact on outcomes in patients with mantle cell lymphoma (MCL) remains poorly characterized. We conducted a nationwide, population-based retrospective longitudinal study using German cancer registry data, including all patients diagnosed with MCL between 2013 and 2023. Regional SES was assessed annually at the district level using quintiles of the German Index of Socioeconomic Deprivation. The primary outcomes were 5-year absolute survival (AS) and relative survival (RS) as a proxy for disease-specific survival. A total of 8871 patients were included. Five-year AS and RS were 62.6% (95% CI, 61.5–63.7) and 72.1% (95% CI, 70.8–73.4), respectively. However, no meaningful differences in AS were observed between patients residing in districts of the lowest and highest SES quintiles (62.0% vs 63.1%). This nationwide population-based study indicates that regional socioeconomic deprivation was not associated with clinically relevant differences in survival outcomes among MCL patients in Germany between 2013 and 2023. These findings differ from previously described patterns in other lymphomas, suggesting that biological disease characteristics may exert a stronger influence on MCL survival than social determinants.

## Introduction

Based on international evidence, low socioeconomic status (SES) has consistently been associated with adverse health outcomes [1–3]. For example, younger and middle-aged adults living in regions with more pronounced socioeconomic deprivation exhibit multimorbidity rates comparable to those of individuals 10–15 years older residing in less deprived regions [4]. This pattern is also evident among cancer patients, as individuals living in socioeconomically deprived areas are disadvantaged with respect to cancer incidence, earlier age of disease onset/diagnosis, and inferior survival outcomes [5–7].

With an incidence of 0.4–1.2 cases per 100,000 persons per year, mantle cell lymphoma (MCL) accounts for approximately 5–7% of all non-Hodgkin lymphoma (NHL) worldwide [8, 9]. MCL is characterized by heterogeneous biological and clinical features, leading to disease courses ranging from indolent presentations to highly aggressive behavior. With a relative 5-year overall survival (OS) of approximately 66%, a substantial proportion of MCL patients die each year from lymphoma-related causes, often after a prolonged and treatment-intensive disease course [8]. Currently, risk stratification in MCL relies on the Mantle Cell Lymphoma International Prognostic Index (MIPI) and its combined variant MIPI-c, which incorporate age, ECOG performance status, serum lactate dehydrogenase levels, leucocyte counts prior to treatment, and optionally the Ki67-proliferation index [10, 11]. Importantly, MIPI and MIPI-c are based solely on disease and patient-related biological factors, while potential non-biological determinants, such as patients’ SES, do not currently influence MCL treatment recommendations.

Recently, the American Society of Hematology’s Health Equity Compendium highlighted the paucity of studies addressing health equity in lymphoma outside the United States (US), noting that only a minority of articles published in *Blood*, *Blood Advances*, or *Hematology, ASH Educational Program* report SES-related data or describe efforts to reduce disparities associated with SES [12]. Correspondingly, the prognostic relevance of SES on survival in MCL patients has been only scarcely investigated, with existing studies remaining limited in scope and generalizability [13–15].

In contrast, substantial SES-related survival disparities have been demonstrated for other B-cell lymphomas. In a prior analysis of a German nationwide, population-based dataset, we observed that patients with diffuse large B-cell lymphoma (DLBCL) residing in low-SES regions experienced significantly worse 5-year OS compared with those residing in middle- and high-SES areas. Since the apparent impact of SES on survival diminished after adjustment for regional premature mortality rates (as a proxy for comorbidity burden), we hypothesized that poorer health-related behaviors and a higher burden of comorbidities among those in socioeconomically deprived areas mainly explain our observations [16]. Similarly, our recent analysis of a large cohort of follicular lymphoma (FL) patients revealed a significant negative association between socioeconomic deprivation and long-term survival of FL patients, particularly among men. After adjusting for additional district-level characteristics and premature mortality, SES-related differences diminished considerably across the overall cohort, particularly among male patients. At the same time, SES-related differences completely disappeared in women, likely reflecting sex-specific differences in comorbidity burden (unpublished results) [17].

Meanwhile, significant progress has been made in the treatment of MCL over recent years e.g., by the incorporation of the Bruton’s-tyrosine-kinase inhibitors in first-line therapies for both younger and older or frail MCL patients [18, 19], the redefining of ASCT for consolidation [18], the rituximab maintenance therapy after induction therapy in younger and older MCL patients [20–22], and the approval of CD19-directed CAR-T-cell therapy for relapsed or refractory MCL [23, 24], underlining the growing importance of equal access to novel therapies. From a political and legal perspective, there are supposedly no differences in access to healthcare services in a universal healthcare system such as Germany’s, which includes compulsory social insurance, legal requirements for preventive measures, and a high standard of medical care. Given our own findings of pronounced SES-related survival disparities in DLBCL and FL, and recognizing the current lack of evidence on efforts to reduce socioeconomic-related disparities in hematology, we aimed to assess the influence of regional SES on long-term survival among MCL patients. To this end, we conducted a comprehensive, registry-based, nationwide population study representing the first of its kind to systematically explore the impact of regional SES on MCL outcomes at a national scale in Germany.

## Materials and methods

### Data sources

This retrospective longitudinal study is based on a validated and harmonized pooled cancer registry dataset provided by the Centre for Cancer Registry Data at the Robert Koch Institute for the years 2013–2023, covering the whole population of Germany with approximately 84 million inhabitants [25]. The Data from the cancer registry is available in different regional resolutions: the 16 federal states at the upper level, corresponding to the Nomenclature des Unités territoriales statistiques (NUTS) hierarchical level 1, and 401 regional districts (territorial status of the year 2020), corresponding to the NUTS-2 level. Furthermore, we extracted aggregated data on the degree of urbanity, as a proxy of availability of specialized care facilities, and premature mortality, as a proxy of comorbidity burden, at the regional level (NUTS-2) from the ongoing spatial observation of the Federal Institute for Research on Building, Urban Affairs, and Spatial Development [26]. Data on regional deprivation at the NUTS-2 level per calendar year are based on the German Index of Socioeconomic Deprivation (GISD) of the Robert Koch Institute [27]. The main analysis was amended by a second data set of the Centre for Cancer Registry Data at the Robert Koch Institute, where also clinical information were available, including the Mantle Cell Lymphoma International Prognostic Index (MIPI: categorized in low risk or intermediate risk and high risk) and therapy approach (categorized in watch and wait and systemic therapy), covering the years 2020-2023 [25]. As this second dataset was less complete for some variables, we used it only for exploratory analyses.

### Patients

We included all patients with a primary diagnosis of MCL coded as “C83.1” and “9673/3” according to the International Statistical Classification of Diseases, German Modification (ICD-10-GM) and ICD-O-3, diagnosed between January 1st, 2013, and December 31st, 2023. Vital status follow-up was available until December 31st, 2024. Exclusion criteria were age at diagnosis below 18 or above 99, and invalid district identifiers. The federal states of Mecklenburg-Western Pomerania, Berlin, Brandenburg, and Saxony-Anhalt were not included due to incomplete death reporting during the study period. Patients with a follow-up duration of less than three months were excluded to remove cases that entered the registry data only from information on death certificates, to avoid immortal time bias, as reports up to three months of the initial report to the cancer registry were employed to specify the diagnosis and to focus on the impact of SES on long-term survival rather than on death due to acute conditions.

### Study variables

At the patient level, study variables included age at diagnosis in years, sex, calendar year of diagnosis (2013–2023) and place of residence, coded according to Germany’s 16 federal states and linked to the district level. SES was measured using the GISD, an area-level deprivation index summarizing aggregated information on employees with a university degree, employees without education, school leavers without qualification, employment rate, unemployment rate, gross earnings, income tax, household income, and debtor quota [27]. The GISD score was linked to each case in the cancer registry dataset by district identifier and calendar year. Based on the distribution of the GISD, SES was grouped into quintiles (high, high-middle, middle, low-middle, and low). Earlier work confirmed the suitability of the index for studying socioeconomic variations in cancer mortality and lymphoma mortality in Germany [6, 16, 28]. Districts were categorized using a spatial typology reflecting their degree of centrality (very central, central, peripheral, very peripheral). Premature mortality (defined as the number of deaths per 1000 residents before age 70 per district) was used as a proxy for the regional comorbidity burden, based on the hypothesis that the general comorbidity burden also affects patients with lymphoma and that this relationship varies by SES [29].

### Outcomes

The primary outcome of the study was death within 5 years following the date of MCL diagnosis. Patients were followed until the date of death, the end date of the study period, or 1827 days (corresponding to 5 years), whichever occurred first.

### Statistical analysis

All analyses were performed using R, version 4.3.2. Categorical variables were summarized as counts and proportions, and continuous variables as medians with interquartile ranges. To compare the effect size of the differences among study groups, standardized mean differences were used, whereby values ≥ 0.10 were interpreted as non-negligible difference. Absolute and relative survival by SES group for each study cohort was estimated based on the age-standardized (International Cancer Survival Standard) [30] complete cohort life-table approach (Ederer II). Associations between SES and 5-year OS were evaluated in multivariable Cox proportional hazards models adjusted for sex, SES group, age at diagnosis, proximity to certified cancer centers, and district-level comorbidity burden. Models incorporated spatial fixed effects for Germany’s 16 federal states to account for time-invariant regional differences, such as quality of cancer registration, and calendar year fixed effects to adjust for temporal confounding. Cluster-robust standard errors at the district level were applied to account for within-district correlation. Results are presented as hazard ratios (HRs) with 95% confidence intervals (CIs). The proportional hazards assumption was evaluated visually using scaled Schoenfeld residuals. As a sensitivity analysis, all models were also applied using transformed time to evaluate the impact of all variables on relative survival. Relative survival is an established proxy for disease-specific survival, enabling the indirect estimation of the disease burden by carefully adjusting for age-, sex-, and calendar year-specific population mortality rates. This approach effectively removes the influence of background mortality, thereby isolating the excess hazard attributable to the disease when explicit cause-of-death data are unavailable [31]. Reporting of the study followed the Strengthening the Reporting of Observational Studies in Epidemiology (STROBE) guidelines [32]. No adjustments for multiple testing were applied, and *p*-values were not reported, as all results represent associations at the population level rather than estimates derived from sampling variability [33].

## Results

**Fig. 1:**
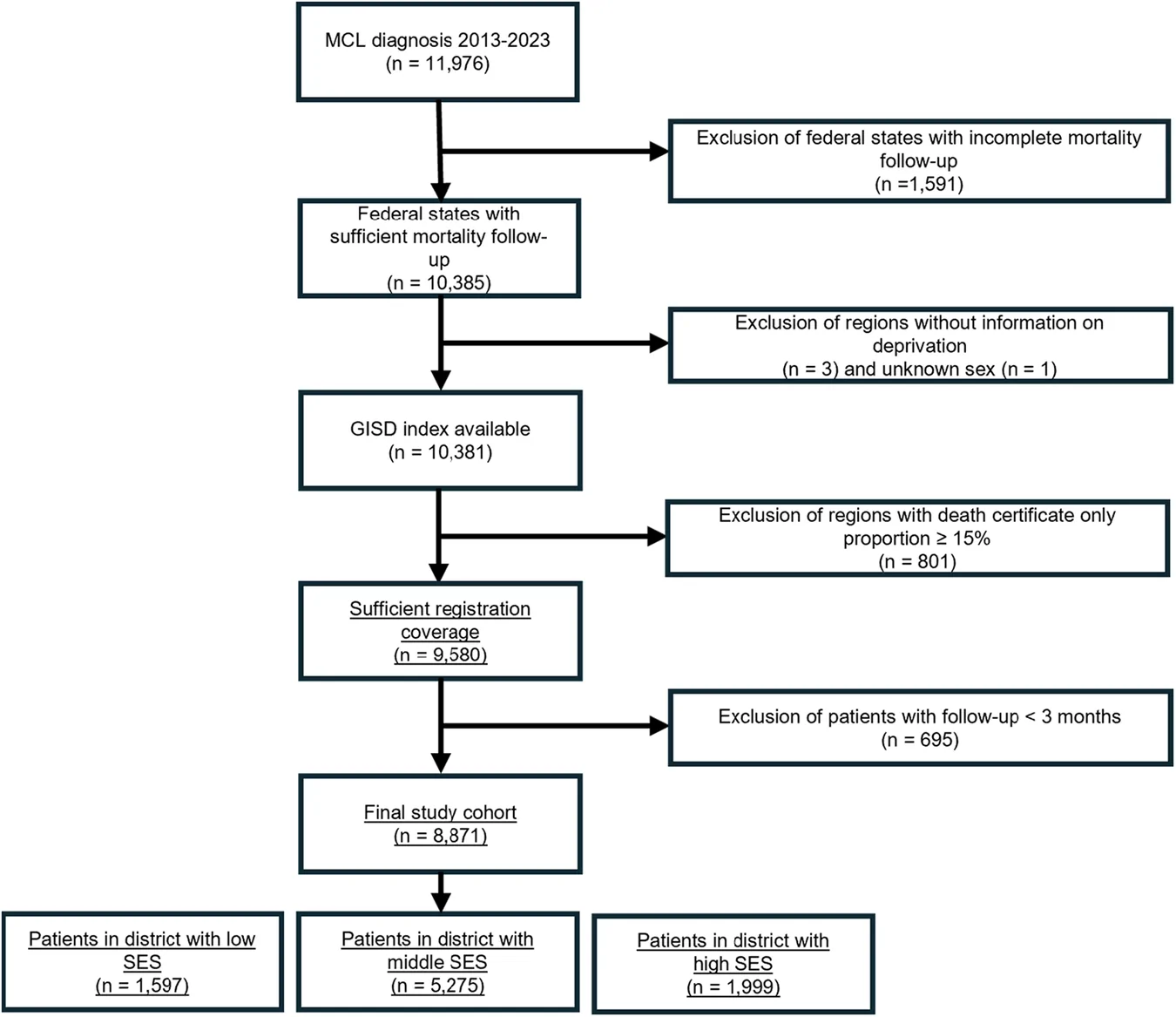
Flow chart of the study cohort. A total of 8871 patients were included in this study.

In total, 8871 patients with a primary diagnosis of MCL diagnosed between 2013 and 2023 (mean age 70.1, 28.9% female, median follow-up 2.6 years) were included in the final analysis (Fig. 1 and Table 1). Of all included patients, 1597 resided in low-SES (deprived) districts, whereas 1999 lived in high-SES districts.

**Table 1:** Baseline characteristics of patients with mantle cell lymphoma by socioeconomic status (epidemiological dataset).

	Socioeconomic status				
	Overall (*N* = 8871)	High (*N* = 1999)	Middle (*N* = 5275)	Low (*N* = 1597)	SMD
Age at diagnosis in years, mean, *n* (%)	70.13(11.3)	70.1 (11.6)	70.2 (11.3)	69.9 (10.9)	0.015
Prior tumor, *n* (%)	311 (42.4)	88 (48.4)	54 (37.0)	52 (40.3)	0.041
Median follow-up in years (Q1, Q3)	3.1 (1.3, 5.00)	3.2 (1.4, 5.00)	3.1 (1.3, 5.00)	3.0 (1.3, 5.00)	0.013
Deaths within 5 years, *n* (%)	3129 (35.3)	707 (35.4)	1873 (35.5)	549 (34.4)	0.016
Female patients, *n* (%)	2566 (28.9)	606 (30.3)	1504 (28.5)	456 (28.6)	0.026
Spatial type, *n* (%)					0.605
Rural sparsely	1130 (12.7)	102 (5.1)	678 (12.9)	350 (21.9)	
Rural densely	1577 (17.8)	198 (9.9)	1121 (21.3)	258 (16.2)	
Urban	4059 (45.8)	885 (44.3)	2694 (51.1)	480 (30.1)	
Larger city	2105 (23.7)	814 (40.7)	782 (14.8)	509 (31.9)	
District level deaths before age 70, per 100.000 persons, median (Q1, Q3)	2.8 (2.5, 3.3)	2.3 (2.1, 2.5)	2.8 (2.6, 3.2)	3.6 (3.3, 3.9)	2.135

Q1: First quartile, Q3: Third quartile, *SMD* Standardized mean difference.

Compared with patients living in high-SES districts, those living in low-SES districts were slightly younger at diagnosis (by approximately 0.2 years), less often female (28.6% vs 30.3%), less likely to have other primary tumors (40.3% vs 48.4%), and lived in districts with higher premature mortality (rate 3.6 vs 2.3, Table 1). Furthermore, patients from low-SES regions were more likely to live in rural sparsely and rural dense regions compared to patients from high-SES regions (21.9% vs 5.1% and 16.2% vs 9.9%, Table 1).

### Survival

**Fig. 2:**
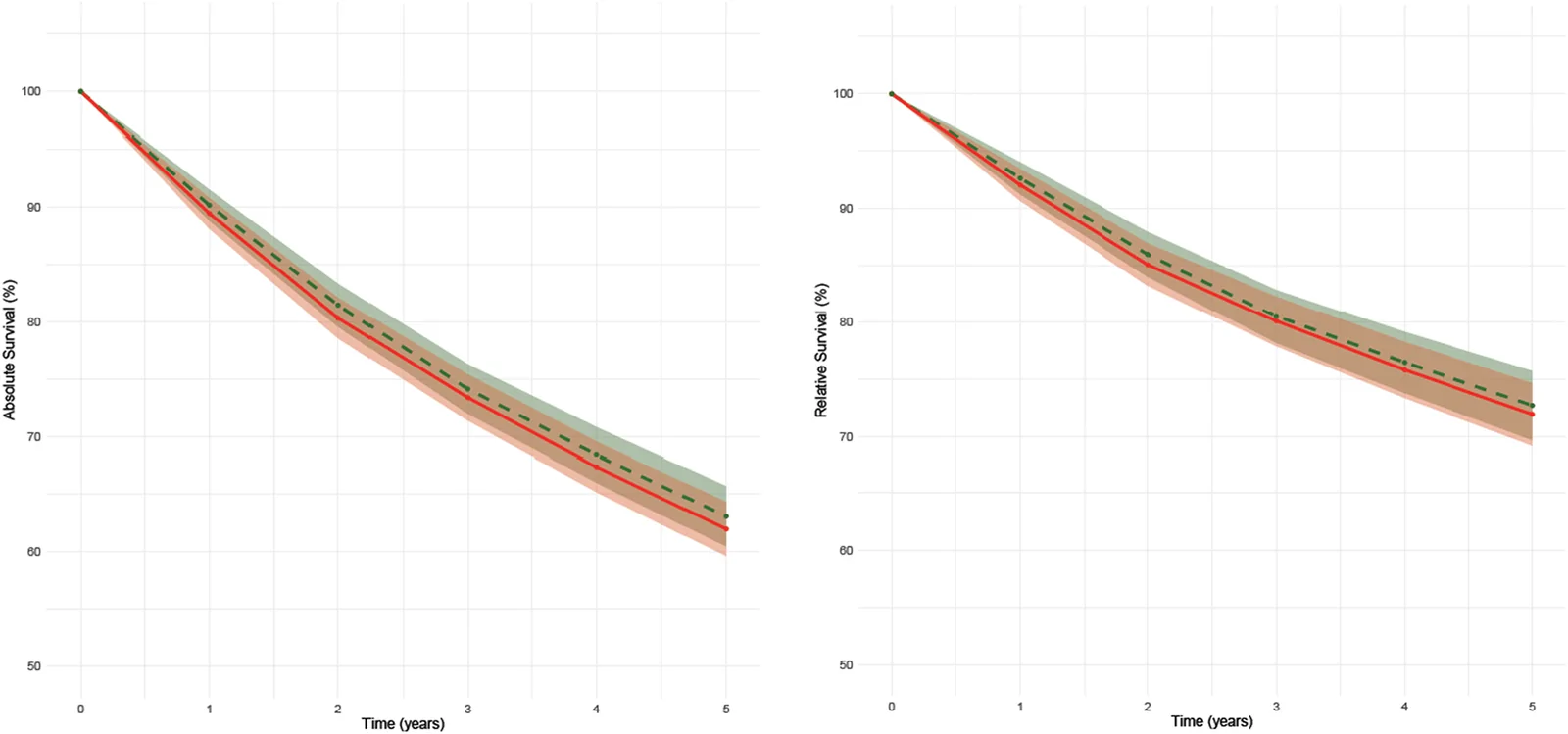
Survival analysis. Age-standardized 5-year absolute survival (left panel) and age-standardized 5-year relative survival (right panel) in patients with mantle cell lymphoma; low socioeconomic status (red solid line) versus high socioeconomic status (green dashed line).

Among MCL patients, 3129 deaths (35.3%) were recorded during the observation period (Table 1), corresponding to a 5-year OS of 62.6% (95% CI, 61.5–63.7). No meaningful differences in 5-year OS were observed between patients living in low- vs high-SES districts (62.0% vs 63.1%). The 5-year RS was 72.1% (95% CI, 70.8–73.4) (Fig. 2).

**Fig. 3:**
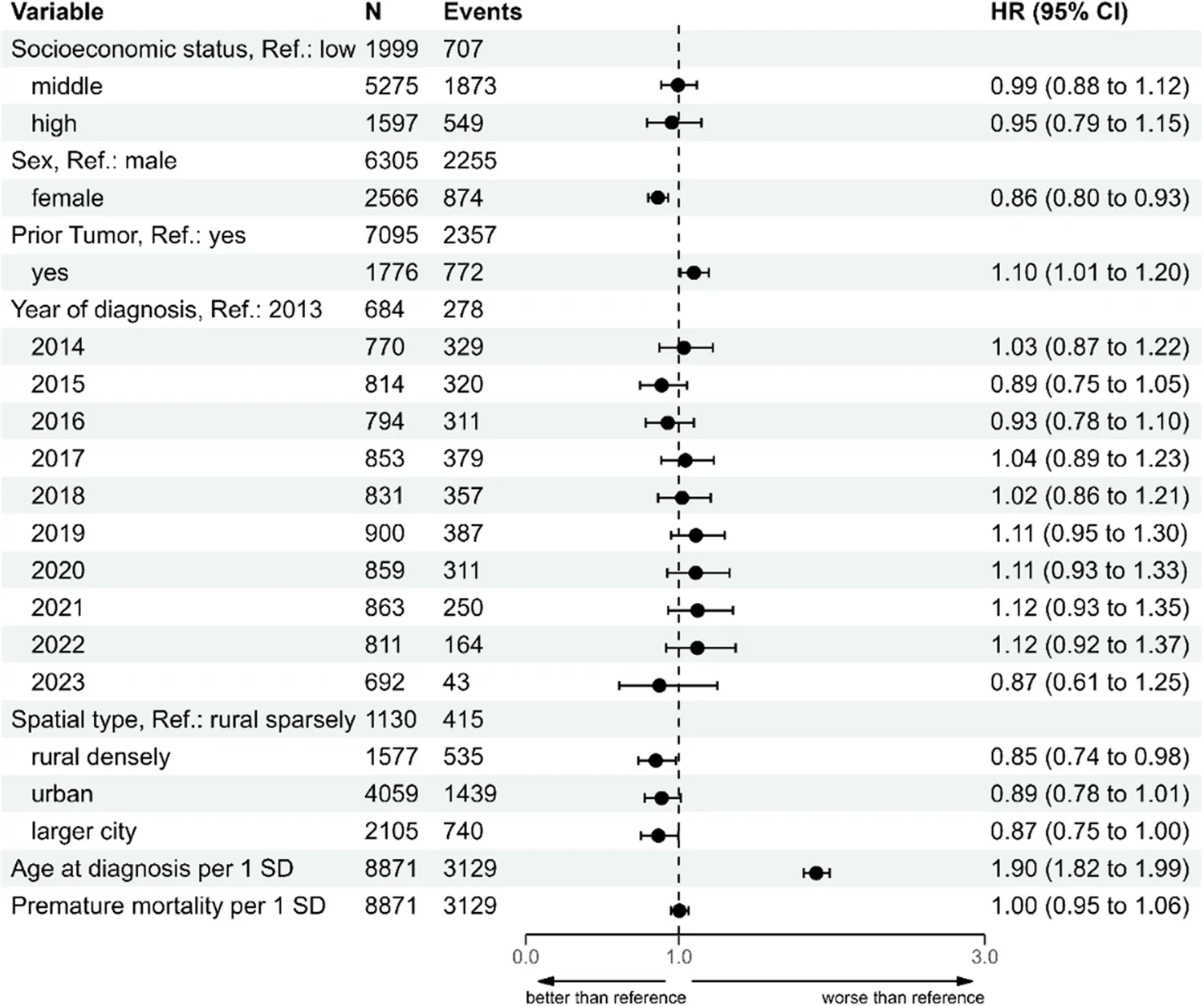
Multivariable Cox proportional hazards model. Predictors of 5-year overall survival in patients with mantle cell lymphoma.

**Fig. 4:**
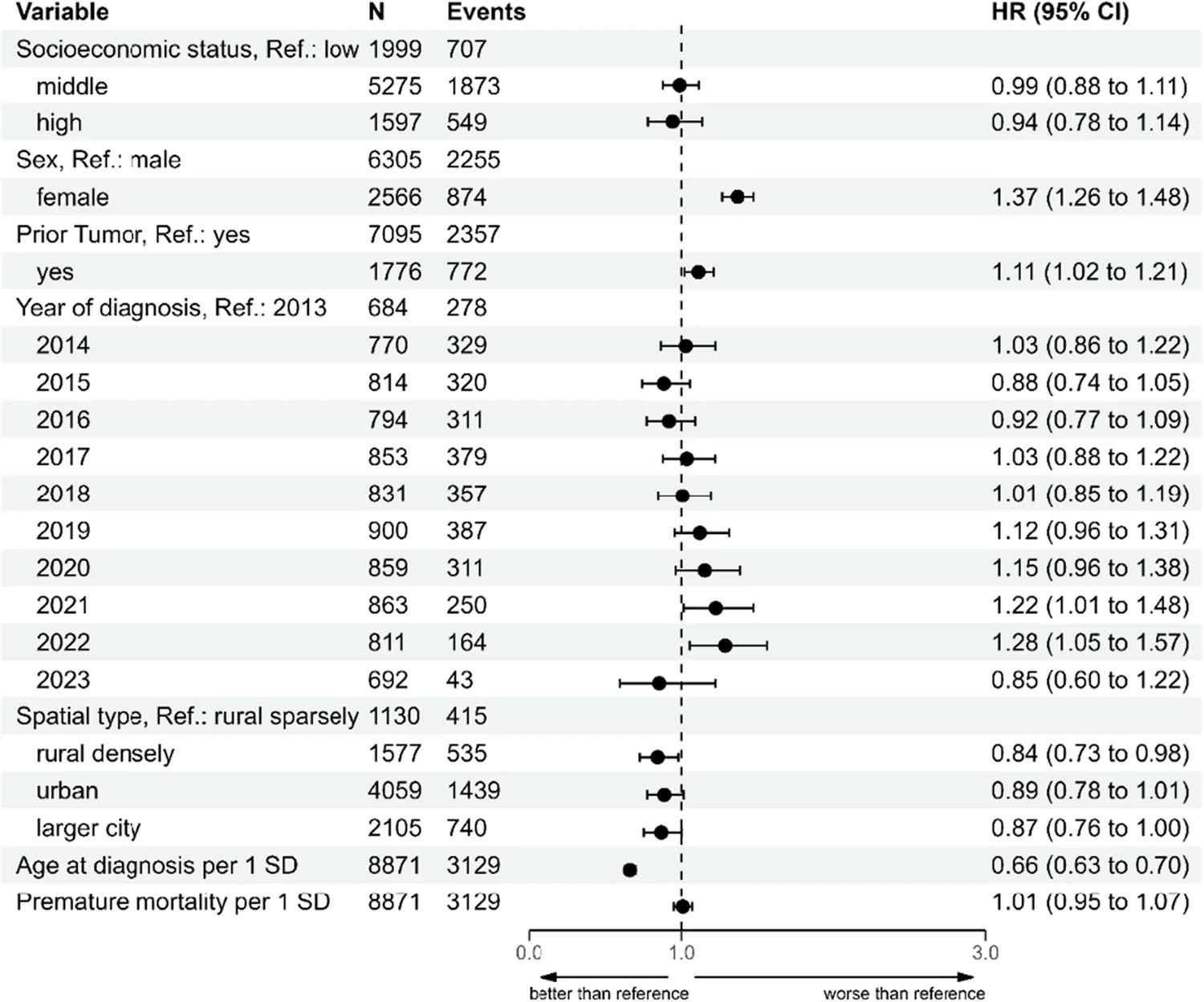
Multivariable Cox proportional hazard models. Predictors of 5-year relative survival in patients with mantle cell lymphoma.

The multivariate Cox regression analyses also indicated an absence of association between SES and overall and relative survival outcomes. The adjusted hazard ratio (HR) for high versus low SES was 0.95 (95% CI, 0.79–1.15) for OS and 0.94 (95% CI, 0.78–1.14) for RS (Fig. 3). As expected, the effect sizes of age and sex were different, with older and male MCL patients showing a higher mortality risk, whereas district-level premature mortality had no influence. In contrast, when analyzing RS as a proxy for disease-specific mortality, females and younger patients appeared more vulnerable (Fig. 4).

Separate analyses of female and male MCL patients showed patterns similar to those observed in the pooled cohort, suggesting that the lack in SES-related differences was consistent across all patient groups.

### Clinical data

The clinical dataset contained 3281 patients with a primary diagnosis of MCL diagnosed between 2020 and 2023 (mean age 70.6, 27.8% female, median follow-up 1.6 years, Table 2). In patients living in low-SES districts, bone marrow was more often affected compared to those living in high-SES districts (21.3% vs 17.0%). Both information on MIPI (share of NA: 82.2% vs 93.1%) and on type of therapy (share of NA: 30.3% vs 33.2%) were more complete in high-SES than in low-SES districts.

**Table 2:** Baseline characteristics of patients with mantle cell lymphoma by socioeconomic status (clinical dataset).

	Socioeconomic status				
	Overall (*N* = 3281)	High (*N* = 798)	Middle (*N* = 1915)	Low (*N* = 569)	SMD
Age at diagnosis in years, mean, *n* (%)	70.61 (11.12)	70.49 (11.85)	70.96 (11.03)	69.64 (10.28)	0.08
Anatomic site					0.078
Bone marrow, *n* (%)	615 (18.7)	136 (17.0)	358 (18.7)	121 (21.3)	
Nodal, *n* (%)	737 (22.5)	191 (23.9)	426 (22.2)	120 (21.1)	
Extranodal, *n* (%)	1930 (58.8)	471 (59.0)	1131 (59.1)	328 (57.6)	
MIPI stage					0.233
Low or intermediate risk	132 (4.0)	52 (6.5)	70 (3.7)	10 (1.8)	
High risk	244 (7.4)	90 (11.3)	125 (6.5)	29 (5.1)	
NA	2906 (88.5)	656 (82.2)	1720 (89.8)	530 (93.1)	
Therapy type					0.098
Watch and wait, *n* (%)	121 (3.7)	37 (4.6)	71 (3.7)	13 (2.3)	
Systemic therapy, *n* (%)	2093 (63.8)	519 (65.0)	1208 (63.1)	366 (64.3)	
NA	1068 (32.5)	242 (30.3)	636 (33.2)	190 (33.4)	
Median follow-up in years (Q1, Q3)	1.63 (0.80, 2.71)	1.71 (0.87, 2.71)	1.63 (0.80, 2.71)	1.49 (0.80, 2.54)	0.069
Deaths up to 5 years, *n* (%)	929 (28.3)	214 (26.8)	572 (29.9)	143 (25.1)	0.071
Female patient, *n* (%)	914 (27.8)	229 (28.7)	522 (27.3)	163 (28.6)	0.021
Median days from diagnoses until systemic therapy (Q1, Q3)	32.0 (16.0, 63.0)	30.0 (14.8, 56.0)	32.0 (16.0, 68.0)	32.0 (18.0, 61.0)	0.032

## Discussion

To date, only a few studies have examined the impact of socioeconomic deprivation on the survival of MCL patients. This study represents the first systematic nationwide investigation to assess the association between socioeconomic deprivation and survival in MCL patients, using a validated multidimensional area-level index integrating indicators of education, employment, and income at the regional level, conducted within a universal health care setting. Contrary to our previous observations in DLBCL and FL [16, 34], we did not observe an adverse impact of low SES on survival among MCL. However, females, independent of their age, had a significantly better OS than males.

The latter finding is in line with a previous study from the US by Wang et al., reporting that female patients suffering from MCL had a significantly better median OS of 76 months compared to male patients with approximately 68 months [35]. However, unlike the few existing studies on the impact of socioeconomic determinants in MCL, which reported comorbidity burden, marital status, type of insurance, educational level, and geographic location as significant prognostic factors, we did not reproduce these associations in our cohort [13, 15]. This may suggest that survival outcomes in MCL are more strongly influenced by biological disease characteristics than by socioeconomic factors. In particular, nodal MCL is associated with a high number of genetic aberrations, marked genetic instability, and an increased mean mutation count in relapsed disease, all of which contribute to clonal evolution [36–38].

Like our previous analysis in DLBCL and FL, this epidemiological study is based on cancer registry data, containing general information on patient characteristics and tumor diagnosis but lacking more specific prognostic variables such as tumor biology and detailed treatment trajectories, as well as lifestyle and comorbidities, but also the economic situation and educational attainment at the individual patient level. Thus, it cannot be fully excluded that SES indeed may exert a considerable prognostic impact on MCL-specific survival if such information were available. However, the potential impact of residual confounding is mitigated by our study design, which explicitly incorporated a range of contextual factors, namely premature mortality as a proxy for lifestyle and comorbidity, and spatial type as a proxy for availability of high-quality treatment. Moreover, by including fixed effects for calendar year and federate state into the models, we indirectly adjusted for unobserved patient characteristics that change over time or differ between districts. Another limitation of the study is that the central conclusions are based only on the single endpoint all-cause mortality. Yet, we also computed the adjusted models using RS as a proxy of disease-specific mortality, which confirmed our main findings.

Taken together, our findings from epidemiological data suggest that, unlike in DLBCL or FL, survival outcomes in MCL may be predominantly shaped by biological disease factors.

This observation reinforces the central role of tumor biology over structural factors, comorbidities, and lifestyle, which may explain why socioeconomic survival gradients observed in other lymphoma entities are largely absent in MCL within a universal healthcare system. Further studies should test this hypothesis based on more detailed clinical and socioeconomic data.

## Data Availability

The data included in the study were analyzed in the protected environment of the Hamburg Cancer Registry. Due to data protection and security regulations, the sensitive patient data cannot be made publicly available. In general, however, data for scientific projects can be requested from the Hamburg Cancer Registry. All analysis scripts can be made available on request from the corresponding author.
